# A fixed-dose 24-hour regimen of artesunate plus sulfamethoxypyrazine-pyrimethamine for the treatment of uncomplicated *Plasmodium falciparum *malaria in eastern Sudan

**DOI:** 10.1186/1476-0711-5-18

**Published:** 2006-08-26

**Authors:** Ishag Adam, Mamoun Magzoub, Maha E Osman, Insaf F Khalil, Michael Alifrangis, Khalid A Elmardi

**Affiliations:** 1 Faculty of medicine, University of Khartoum, Sudan; 2 University of Kassala, Sudan; 3 National Center for Research, Sudan; 4 Department of International Health, Institute for Medical Microbiology and Immunology, Center for Medical Parasitology (CMP), Copenhagen; 5 National Malaria control, Ministry of Health, Sudan; 6 The Academy of Medical Sciences and Technology, Khartoum, Sudan

## Abstract

**Background:**

Artemisinin-based combination therapy is increasingly being adopted as first-line antimalarial therapy. The choice of appropriate therapy depends on efficacy, cost, side effects, and simplicity of administration.

**Methods:**

the efficacy of fixed co-formulated (f) artesunate-sulfamethoxypyrazine-pyrimethamine (AS+SMP f) administered at time intervals of 12 hours for a 24-hour therapy was compared with the efficacy of the same drug given as a loose combination (AS+SMP l) with a dose interval of 24 hours for 3 days for the treatment of uncomplicated *Plasmodium falciparum *malaria in eastern Sudan.

**Results:**

seventy-three patients (39 and 34 in the fixed and the loose regimen of AS+SMP respectively) completed the 28-days of follow-up. On day 3; all patients in both groups were a parasitaemic but one patient in the fixed group of AS+SMP f was still febrile.

Polymerase chain reaction genotyping adjusted cure rates on day 28 were 92.3% and 97.1% (*P *> 0.05) for the fixed and loose combination of AS+SMP respectively.

Three (4.1%) patients (one in the fixed and two patients in the loose group of AS+SMP) in the study suffered drug-related adverse effects.

Gametocytaemia was not detected during follow-up in any of the patients.

**Conclusion:**

both regimens of AS+SMP were effective and safe for the treatment of uncomplicated *P. falciparum *malaria in eastern Sudan. Due to its simplicity, the fixed dose one-day treatment regimen may improve compliance and therefore may be the preferred choice.

## Background

There are almost 515 million episodes of clinical *Plasmodium falciparum *malaria infections [1]. Malaria treatment and control have been undermined by emergence and spread of drug-resistant malaria worldwide hereby increasing morbidity and mortality. World Health Organization's strategies to reduce malaria-related mortality depend on early diagnosis and effective treatment with an appropriate drug and now artemisinin-based combination therapies (ACTs) are recommended [2,3]. Malaria causes up to 7.5 – 10 million cases and 35000 deaths every year in Sudan [4]. Due to the spread of multi-drug resistant *Plasmodium falciparum *malaria in Sudan [5], artesunate plus sulfadoxine-pyrimethamine (AS+SP) is at this moment the recommended first-line treatment for uncomplicated *P. falciparum *malaria.

The choice of an ACT in specific malaria-endemic areas needs to take into account their efficacy, side effects, price and mode of administration. Common ACT therapies last for 3 days and in some instances the patient has to swallow up to 24 tablets. Sulfamethoxypyrazine (SM) is chemically very close to sulfadoxine but has different pharmacokinetics [6,7]. The high efficacy of sulfamethoxypyrazine-pyrimethamine (SMP) for treating *P. falciparum *malaria had been demonstrated previously [8-10]. It is expected that the combination of this drug with artesunate will result in a highly efficacious treatment for *P. falciparum *malaria. The manufacturer's (Dafra Pharma, NV) accomplished this combination and new treatment was considered for use as a single dose per day over 3 days. According to the pharmacokinetics property [6,7], it was explored it would be possible to reduce the dosing interval between each tablet to 12 h and therefore limit the treatment duration to 24 hours. The objective of this trial was to investigate the efficacy of this drug (AS+SMP as two different regimens) in the treatment of uncomplicated *P. falciparum *malaria, since no published data exists yet concerning these drug combinations.

## Patients and methods

The study was conducted in September-November 2005 at AL Sawagi ALganoubia health center in Kassala, eastern Sudan. The area is characterized by low malaria transmission [11]. Febrile (temperature ≥ 37.5°C) patients with uncomplicated *P. falciparum *malaria [12] andno history of antimalarial drug use during the preceding two weeks were recruited for the study. Pregnant women and patients with mixed infections were excluded.

After obtaining informed consent from the patient or the child's parents, a fixed questionnaire including relevant socio-demographic characteristics, medical history, physical findings and investigations conducted was completed for each patient.

### Laboratory methods

Blood films were prepared, Giemsa-stained and examined by 100× oil immersion fields. The parasite density was counted against 200 leucocytes, assuming 8000 leucocytes/μl. All slides were double-checked blindly and only considered negative if no parasites were detected in 100 oil immersion fields. If gametocytes were seen, then the count was extended to 500 leucocytes.

Three spots of blood were taken on filter-paper initially and later if parasites reappeared microscopically during the follow-up period to differentiate between re-infection and recrudescence using nested PCR where FC 27 and ICI alles of Primers from polymorphic *P. falciparum *merozoite surface protein 1&2 (MSP1 & MSP2) were detected [13,14].

### Treatment and follow up

The manufacturers' (Dafra Pharma NV) instructions were strictly followed. The patients were alternatively allocated either to the fixed or the loose combination of AS+SMP. The patients appointed to the fixed dose group were given 3 doses of AS+SMP f orally as a fixed combination (one single tablet every 12 hours (a total treatment lasted 24 hours). The fixed dose tablets were available in three different dosage strengths, each one containing AS/SM/P mg in different doses; 200/500/25 for adults (≥40 kg), 100/250/12.5 for children and adolescents (16–39.9 kg) and 50/125/6.25 for babies and infants (≤15.9 kg).

The patients in the group receiving the loose combination were also given the treatment according to their weight. They received two different tablets which were colour-coded for ease of administration: pink SMP tablets and white AS tablets. Their treatment regimen lasted for three days with intervals of 24 hours.

The tablets were crushed and dissolved in water for children who were not able to swallow them. Subjects were observed for vomiting for one hour; the full dose was repeated for those who vomited within 30 min and half of the dose was repeated if vomiting occurred between 30 and 60 minutes.

### Follow-up and re-treatment

Patients were requested to come back on days 1, 2, 3, 7, 14, 21 and 28 and at any time they felt unwell. At each visit, body temperature was measured and blood films were prepared. Haemoglobin was measured on day 0 and day 28. During the follow-up the patients were asked if they suffered from side effects which could be expected from the treatment (nausea, vomiting, abdominal pain, dizziness and rash); these symptoms were considered to be drug related if they had not been reported at the patient's first presentation to the clinic.

The second-line treatment for uncomplicated *P. falciparum *malaria in Sudan (Artemether-lumfantrine) was given for treatment failures and the patients were followed as above. Classification was done based on the following: Early Treatment Failures (ETF) in case of significant parasitaemia at day 2 or 3 or parasites and fever at day 3, Late Clinical Failures (LCF) for cases with parasites and fever during follow-up after day 3 and Late Parasitological Failures (LPF) for parasite infections with/without fever during the follow-up period. Cases which remained negative during follow-up were considered to be Adequate Clinical and Parasitological Responses (ACPR). These were modified WHO guidelines [12,15].

### Statistics

Data were entered into a computer database and SPSS software (SPSS Inc., Chicago, IL, USA) was used for statistical analysis. The means (age, weight, temperature, haemoglobin and parasite count) were calculated for all the patients and were compared between the patients in the two arms of the study using student t-test, when the data was normally distributed and by Mann-Whitney test if the data was not normally distributed. Percentages were calculated and compared for the patients by X^2 ^test. *P *< 0.05 was regarded significant.

### Ethics

The study received ethical clearance from the Ethical board of the faculty of medicine, University of Khartoum.

## Results

**Figure 1:**
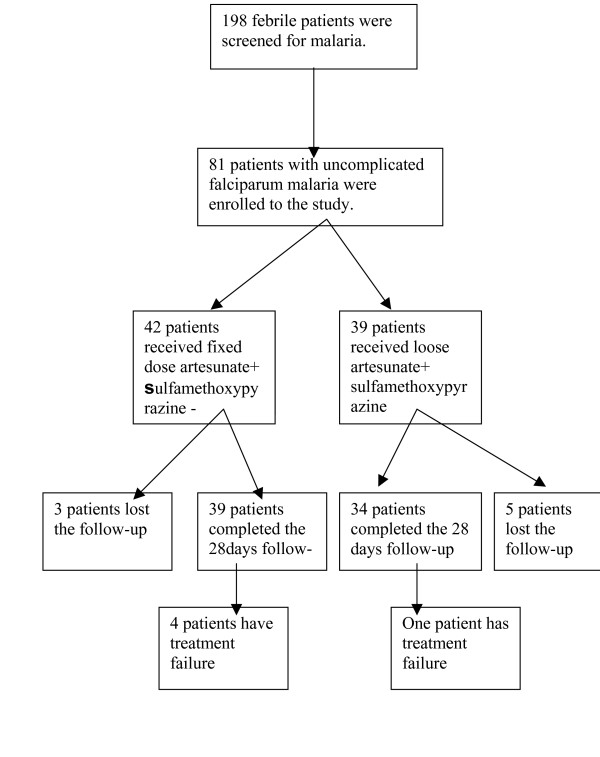
Trial profile, showing number of patients enrolled, treated and completing the 28 days of follow-up after the treatment with fixed or loose combinations of AS+SMP

During the period of the study, eighty-one patients presented with uncomplicated *P. falciparum *malaria. Seventy-three of them fulfilled the selection criteria and completed the 28-days follow-up; 39 and 34 in the fixed and the loose combinations of AS+SMP groups respectively. Eight patients, three in the fixed and five in the loose group were lost during the follow-up period because they changed their addresses, figure 1.

Nine (12.3%) patients were children under five years of age; 5 (12.8%) vs. 4 (11.8%) of them in the fixed and the loose group of AS+MSP respectively. There was no significant difference in the presenting age, weight, temperature, parasite count and the haemoglobin level between the two groups, table 1.

**Table 1:** The base line (day 0) characteristic of the 73 patients who completed the 28 days of follow-up after the treatment with the fixed or loose combinations of AS+SMP

	*Fixed AS+SMP Group*	*Loose AS+SMPGroup*
*No. of patients*	39	34
*MEAN VALUE AND (SD) For*.		
*Age (years)*	15.1 (12.5)	17.6 (11.1)
Weight (Kg)	33.8 (20.04)	39.8 (21.3)
Temperature (°C)	38.4 (0.8)	38.1 (0.93)
Parasite count (rings/μl)	18680.2 (26458.4)	21342.06 (23371.8)
Haemoglobin (gm/dl)	11.2 (1.5)	11.5 (1.50)

Three (4.1%) patients (one in the fixed dose and two in the loose group of AS+SMP) in the study suffered drug-related adverse effects (nausea, vomiting, diarrhea, itching and dizziness), *P *> 0.05. However, these were mild and resolved spontaneously.

On day 1; four (10.3%) vs. one (2.9%) patient was still febrile, *P *> 0.05 and one (2.6%) was parasitaemic in the fixed group of AS+SMP.

On day 3; one patient in the fixed group of AS+SMP was still febrile but all the patients in both groups were aparasitaemic.

By day 28, four (10.3%) and one (2.9%) patient (*P *= 0.36) in the fixed and the loose group of AS+SMP respectively, showed late treatment and parasitological failures, the rest (89.7% vs. 97.1%) of the patients had an adequate treatment response.

The results of parasite genotyping indicated that four (one is the patient of loose and three patients in the fixed group of AS+MSP) of these five treatment failures were cases of recrudescence, where the parasites collected from each relapse parasitaemia appeared to be identical, in terms of their MSP1 & MSP2, to those collected pretreatment from the same patients. The fifth relapse in the fixed group (day 28) was caused by re-infection, where the parasite alleles were different.

So PCR corrected cure rates were 92.3% and 97.1% (P > 0.05) for the two groups respectively.

At the beginning of the study, gametocytaemia was detected in three patients (two in the fixed group), and it was not detected during the follow-up in any of the patients

## Discussion

This is probably the first publication on efficacy of AS+SMP in the treatment of uncomplicated falciparum malaria. The high cure rate demonstrated for AS+-SMP (>90%) in this study was expected because of the synergistic effect of artesunate and SMP and potentially because of the non-wide use of SMP as an antimalarial in this country. Previously high efficacy of SMP for the treatment of uncomplicated *P. falciparum *malaria was reported in other African countries [8-10].

Although the loose combinations of AS+SMP was slightly (not significantly) more efficacious than the fixed one, the fixed regimen of AS+SMP is most promising since it permits reduction of the treatment duration to a maximum of 24 h. The mechanism of action of SM drug is the same as of sulfadoxine(S), but its protein binding and elimination half-life permit the new regimen to be developed in SM rather than in SP [6,7,16]. The repeated loading dose of AS is responsible for a drastic destruction of parasites whereas the SMP combination enables to protract the favorable effect, by continued inhibition of folic acid biosynthesis in the parasite to over 15 days. The intake of only three tablets, in combination with the short duration of the treatment may be of paramount importance as an influence on the patients' compliance. The single dose of SP was reported to have the best compliance among antimalarial tested in Sudanese patients [17]. The treatments with artemether + lumefantrine and AS + amodiaquine (the therapies recommended by the WHO) suffer from the large number of tablets (>20) and this may influenced the compliance adversely. The fixed short interval regimen of AS+SMP seems to be simple, effective and may enhance the compliance. Yet, two parasite asexual life-cycles need to be exposed to the artemisinin derivative to maximize the reduction in parasite numbers and thereby minimizing the opportunity for resistance selection while providing optimum therapeutic responses [18]. However, single high dose administration of 300 mg artesunate as one dose, followed by mefloquine on day 24 and 30 h, resulted in a 97% cure rate of malaria patients in Thailand [19], which indicates that short treatment regimens of artemisinin-derivatives with appropriate combinations are effective.

In our study, the cure rates were 92.3% and 97.1 (P > 0.05) for both arms respectively. Treatment failures – even, AS+SP treatment failure – has recently been reported in the same area of the study [20]. Fewer mild side effects were reported in both arms of the study, this itself might improve the compliance of the patients towards the treatment. Furthermore, these adverse effects were mild and were not different from the adverse effects reported with AS+SP in eastern Sudan [20,21]. Recently, high cure rates and minimum adverse effects were reported among patients with uncomplicated falciparum malaria treated with SMP [10].

There was no gametocytaemia during the follow-up period. High levels of gametocytaemia had been reported in the eastern Sudan following non-artemisinin antimalarials [22], but it had not been reported following ACTs [21]. The ability of artesunate to reduce the post-treatment gametocytaemia is important, as it may reduce transmission [23].

## Conclusion

This was a small-sized study, larger size studies are urgently needed to investigate the efficacy of this new formulated drugs combinations. Both regimens of AS+SMP were effective and safe for the treatment of uncomplicated *P. falciparum *malaria in eastern Sudan. Due to its simplicity the fixed dose one-day treatment regimen may improve compliance and therefore may be the preferred choice

## Authors' contributions

IA, MM, MEO carried out the study and participated in the statistical analysis and procedures. IFK, MA participated in the genotyping of the parasite. IA coordinated and participated in the design of the study, statistical analysis and the drafting of the manuscript. All the authors read and approved the final version.
